# Metabolic Disruptions in Zebrafish Induced by α-Cypermethrin: A Targeted Metabolomics Study

**DOI:** 10.3390/toxics13070529

**Published:** 2025-06-24

**Authors:** Hang-Ji Ok, Ji-Woo Yu, Jung-Hoon Lee, Eun-Song Choi, Jong-Hwan Kim, Yoonjeong Jeon, Won Noh, Sung-Gil Choi, Jeong-Han Kim, Min-Ho Song, Ji-Ho Lee

**Affiliations:** 1 Department of Agricultural Biotechnology of Agriculture and Life Sciences, Seoul National University, Seoul 08826, Republic of Korea; hjok@shimadzu.co.kr (H.-J.O.); kjh2404@snu.ac.kr (J.-H.K.); 2 Department of Technical Research Center, Shimadzu Scientific Korea, Seoul 08506, Republic of Korea; 3 Department of Crop Science, Konkuk University, 120 Neungdong-ro, Gwangjin-gu, Seoul 05029, Republic of Korea; uzu1030@gmail.com; 4 School of Natural Resources and Environment Science, College of Agriculture and Life Sciences, Kangwon National University, Chuncheon 24341, Republic of Korea; elee4128@naver.com (J.-H.L.); dmsthd0301@naver.com (E.-S.C.); 5 Department of Food Biotechnology and Environmental Science, Kangwon National University, Chuncheon 24341, Republic of Korea; 6 Environmental Safety Research Center, Korea Institute of Toxicology (KIT), Jinju 52834, Republic of Korea; jjong@kitox.re.kr (J.-H.K.); yoonjeong.jeon@kitox.re.kr (Y.J.); won.noh@kitox.re.kr (W.N.); sgil@kitox.re.kr (S.-G.C.); 7 Human and Environmental Toxicology Program, Korea University of Science and Technology (UST), Deajeon 34113, Republic of Korea

**Keywords:** metabolomics, α-cypermethrin, zebrafish, metabolic changes

## Abstract

The widespread application of pesticides in agriculture has raised increasing concerns regarding their ecological impact, particularly in aquatic environments. Among these, α-cypermethrin, a highly active isomeric form of cypermethrin, has been extensively used due to its potent insecticidal efficacy and low mammalian toxicity. However, its toxicity to non-target aquatic organisms remains insufficiently understood at the metabolic level. In this study, a targeted metabolomics approach was employed to investigate the biochemical effects of α-cypermethrin in adult zebrafish. Acute toxicity was first determined to establish sublethal exposure concentrations (0.15 µg/L and 1.5 µg/L), followed by a 48 h exposure under a controlled flow-through system. GC-MS/MS-based analysis quantified 395 metabolites, and multivariate statistical models (principal component analysis (PCA) and partial least square-discriminant analysis (PLS-DA)) revealed clear dose-dependent metabolic alterations at two time points. Pathway analysis identified disruptions in glycolysis, glycerolipid metabolism, amino acid turnover, and glutathione pathways. Notably, glutamate depletion and associated reductions in GABA (4-Aminobutanoate) and TCA (Tricarboxylic acid) cycle intermediates suggest oxidative stress-induced metabolic bottlenecks. These results provide mechanistic insights into α-cypermethrin-induced toxicity and demonstrate the utility of metabolite-level biomarkers for environmental monitoring. This study contributes to a systems-level understanding of how sublethal pesticide exposure affects vertebrate metabolism, offering a basis for improved ecological risk assessment and pesticide regulation.

## 1. Introduction

Pesticides are extensively employed in modern agriculture to enhance crop productivity, minimize post-harvest losses, and control pests that spread disease [1,2]. However, the increasing scale of pesticide application has resulted in substantial environmental dissemination, raising significant concerns regarding ecological safety [3,4,5,6,7,8]. Notably, less than 0.3% of applied pesticides effectively reach their intended targets, while the majority are dispersed into surrounding soil and aquatic systems [9,10]. These chemicals subsequently leach into groundwater or are transported via surface runoff into adjacent water bodies, causing the unintended exposure of aquatic organisms to residual toxicants [1,11].

Cypermethrin is a widely used insecticide in both farming and pest control due to its strong ability to kill insects while posing a relatively low toxicity threat to mammals [12,13,14]. One of its most potent forms, α-cypermethrin, is enriched with the most active isomers, enhancing its effectiveness [12]. This formulation has become especially popular in countries such as India, China, and Pakistan for protecting crops [13,14]. In India, for example, production rose dramatically from 6.5 metric tons in 2005 to more than 2400 metric tons by 2010, illustrating a rapid rise in use over recent years [13].

The growing use of α-cypermethrin has raised environmental concerns, as residues are often found in surface waters, sediments, and agricultural runoff [13,14,15]. Even at low concentrations, α-cypermethrin has been shown to cause neurotoxicity, oxidative stress, and abnormal behavior in aquatic organisms [15,16]. In zebrafish and other freshwater species, exposure leads to symptoms such as uncoordinated swimming, balance loss, swelling around the heart, and spinal deformities [17,18,19,20,21]. Although many toxic effects have been reported, the exact molecular mechanisms behind these physiological changes are still not fully understood.

Traditional toxicological assessments often rely on observable phenotypic endpoints, potentially overlooking early or subtle biochemical alterations induced by chemical exposure [22]. Metabolomics analyzes small molecules in living systems to give a clear picture of their biological state [23,24,25]. It can detect subtle changes caused by environmental stressors, including toxic chemical exposure [26,27]. This makes it a useful approach for identifying key disruptions in metabolism and understanding how toxic effects develop at the molecular level [28,29]. Targeted metabolomics was employed to ensure high sensitivity and quantification accuracy, particularly for metabolites involved in oxidative stress and energy metabolism. Such targeted approaches are especially valuable in sublethal exposure studies where subtle metabolic disruptions are expected.

Zebrafish (*Danio rerio*) are widely employed as a model organism in ecotoxicological studies for evaluating the toxicity of aquatic contaminants [30,31,32]. Their short life cycle, high fecundity, and optical transparency during early developmental stages provide practical advantages, making them an essential vertebrate model in environmental toxicology [32]. In the present study, a targeted metabolomics approach was applied to investigate the toxicological effects of α-cypermethrin exposure in adult zebrafish. The study also aims to identify metabolite-level biomarkers that are sensitive indicators of toxic stress and can be utilized for environmental monitoring and ecological risk assessment.

## 2. Materials and Methods

### 2.1. Materials

α-Cypermethrin, used for zebrafish toxicity testing, and ribitol used as internal standard were purchased from Sigma-Aldrich (St. Louis, MO, USA). Methoxyamine hydrochloride, N-methyl-N-(trimethylsilyl)trifluoroacetamide reagent containing 1% trimethylsilyl chloride (MSTFA), and pyridine for derivatization were produced by the same supplier. Methanol, distilled water, and chloroform for sample extraction were obtained from Samchun chemicals (Pyeongtaek, Republic of Korea).

### 2.2. Experimental Fish

Zebrafish were obtained from the fish culture laboratory at the Korea Institute of Toxicology (KIT, Jinju, Republic of Korea) and used for exposure experiments. A total of 200 individuals were acclimated for two weeks in glass aquaria containing dechlorinated tap water (23.7 ± 0.1 °C), under a 16:8 h light/dark photoperiod controlled by an automatic timer. The exposure temperature and photoperiod were intentionally selected to mimic realistic environmental conditions rather than optimal laboratory settings, allowing for a more ecologically relevant interpretation of the sublethal effects. During the acclimation period, the fish were fed once daily with a commercial diet. After acclimation, the fish were randomly assigned to either the solvent control group or one of the α-cypermethrin treatment groups.

### 2.3. Acute Toxicity Assessment of α-Cypermethrin in Zebrafish

The acute toxicity of α-cypermethrin was evaluated using a continuous flow-through system (Hanalab Corp., Daejeon, Republic of Korea), in accordance with OECD Guideline No. 203 [33]. The assay employed five concentrations of α-cypermethrin (0.125, 0.25, 0.5, 1.0, and 2.0 µg/L), each tested in duplicate. Experimental conditions were maintained at 23.7 ± 0.1 °C, with dissolved oxygen levels of 7.9–8.7 mg/L and pH values ranging from 7.4 to 8.1. Two types of control groups were included: a dilution water control and a solvent control (acetonitrile).

Stock solutions of α-cypermethrin (1.25–20.0 mg/L) were prepared in acetonitrile and delivered into a mixing chamber at a flow rate of 10 µL/min using a syringe pump. Simultaneously, dilution water was supplied at 100 mL/min with continuous stirring to ensure uniform mixing. Each exposure vessel contained seven zebrafish and was assigned to a specific test group.

Mortality and sublethal effects were assessed at 24, 48, 72, and 96 h post-exposure. A 16:8 h light/dark photoperiod was maintained throughout the exposure period. Neither feeding nor additional aeration was provided during this time. The median lethal concentration (LC_50_) and its associated 95% confidence interval were estimated using the moving average angle method.

### 2.4. Chemical Exposure and Sample Collection

To ensure stable exposure concentrations, a continuous flow-through system was employed. A solvent control tank (containing acetonitrile in dilution water) and two treatment tanks with different concentrations of α-cypermethrin were set up. All components in contact with the test solutions, including tanks, mixing chambers, and tubing, were made of glass and teflon to minimize the adsorption of α-cypermethrin. α-Cypermethrin stock solutions and dilution water were delivered at flow rates of 10 µL/min and 100 mL/min, respectively, thus achieving a final dilution ratio of 1:10,000. This setup yielded target concentrations of 0.15 µg/L and 1.5 µg/L in the treatment groups, respectively. Each tank, including both control and treatment groups, contained 20 zebrafish. At 24 and 48 h post-exposure, six fish per group were randomly sampled for subsequent analyses.

### 2.5. Water Sample Analysis

Water samples were analyzed using a UHPLC–MS/MS system (LCMS-8060NX, Shimadzu, Kyoto, Japan). The UHPLC unit was equipped with a degasser (DGU-405), a solvent delivery module (LC-40D X3), an autosampler (SIL-40C X3), and a column oven (CTO-40C). Chromatographic separation was performed on a Shim-pack GISS C18 column (2.1 × 50 mm, 1.9 µm; Shimadzu), maintained at 40 °C. The injection volume was 50 µL. A binary gradient elution was employed using mobile phase A (5 mM ammonium acetate with 0.1% formic acid in water) and mobile phase B (5 mM ammonium acetate with 0.1% formic acid in methanol). The flow rate was maintained at 0.3 mL/min. The gradient program was as follows: 95% A/5% B was held for 0.5 min, then linearly adjusted to 40% A/60% B at 1.5 min. The mobile phase was further shifted to 100% B at 6.0 min and maintained until 8.0 min. The system was subsequently re-equilibrated to the initial conditions (95% A/5% B) at 8.1 min and held for 1.9 min.

The mass spectrometer was operated in positive electrospray ionization (ESI) mode. Argon was employed as the collision-induced dissociation (CID) gas, and quantification was performed in multiple reaction monitoring (MRM) mode. MRM transitions were optimized based on full-scan spectra. For each analyte, three product ions were selected based on their fragmentation patterns: one was used for quantification and two for confirmation. Appropriate collision energies were applied for each transition. Instrument control and data analysis were conducted using LabSolutions software (version 5.128 SP1, Shimadzu). Detailed MRM parameters and instrument settings are summarized in Tables S1 and S2.

### 2.6. Sample Preparation for Metabolomics Analysis

Individual zebrafish samples were homogenized using a cryogenic grinding system (Freezer Mill 6875, SPEX SamplePrep™, Metuchen, NJ, USA). The protocol consisted of a 15 min pre-cooling step, followed by three grinding cycles, each comprising 2 min of grinding and 1 min of intermediate cooling. The total grinding processing time was approximately 23 min per sample.

Homogenized tissue samples (50 mg) were extracted with 500 μL of methanol containing ribitol (5 μg/mL) as an internal standard. After shaking at room temperature for 5 min, 250 μL of distilled water was added, followed by vortexing for 1 min. The mixture was centrifuged at 14,000× *g* for 5 min at 4 °C, and 600 μL of the resulting supernatant was transferred to a new tube. Subsequently, 400 μL of chloroform was added, vortexed for 1 min, and centrifuged again at 14,000× *g* for 15 min at 4 °C. A 200 μL aliquot of the upper phase was collected and dried using a vacuum concentrator (Modulspin 40, Hanil Scientific, Gimpo, Republic of Korea). The dried extract was derivatized by adding 50 μL of methoxyamine hydrochloride solution (20 mg/mL in pyridine) and incubating at 37 °C for 90 min. This was followed by trimethylsilylation with 50 μL of MSTFA at 37 °C for 30 min.

### 2.7. Instrumental Conditions for Targeted Metabolites Using GC-MS/MS

Metabolite profiling was performed using a Shimadzu GCMS-TQ8050 instrument (Kyoto, Japan) operated in multiple reaction monitoring (MRM) mode, targeting a total of 395 metabolites. Among these, 331 metabolites were identified using Shimadzu’s Smart Metabolites Database, while the remaining 64 were annotated based on an in-house library.

Each sample (1.0 μL) was injected in split mode (40:1) into a BPX-5 capillary column (30 m × 0.25 mm i.d., 0.25 μm film thickness; TRAJAN). The injector, ion source, and transfer line temperatures were set to 250 °C, 200 °C, and 280 °C, respectively. The oven temperature was initially held at 60 °C for 2 min, ramped at 10 °C/min to 320 °C, and maintained at this temperature for 15 min. Helium was used as the carrier gas at a constant flow rate of 1 mL/min, and argon was used as the collision gas. Electron ionization was performed at 70 eV.

Data acquisition and processing were carried out using GCMSsolution software (version 4.3, Shimadzu). A manual verification of peak identification was performed, and metabolite abundances were calculated as peak area ratios relative to the internal standard ribitol.

### 2.8. Statistical Analysis

Multivariate statistical analyses were conducted using MetaboAnalyst 6.0 (www.metaboanalyst.ca, accessed on 12 May 2025), a web-based platform for metabolomics data interpretation. Unsupervised principal component analysis (PCA) was used to evaluate clustering patterns between the control and treatment groups, while supervised partial least squares-discriminant analysis (PLS-DA) was applied to enhance group separation. Model quality was assessed based on R^2^ (goodness of fit) and Q^2^ (predictive ability) values.

Univariate statistical analysis was performed using a *t*-test with a significance threshold of *p* < 0.05. A hierarchical clustering heatmap was generated based on the relative abundances of selected biomarker metabolites identified through the *t*-test. Metabolic pathway analysis was also conducted in MetaboAnalyst 6.0, with pathway identification referenced to the *Danio rerio* KEGG pathway library.

## 3. Results and Discussion

### 3.1. Determination of Acute Toxicity of α-Cypermethrin in Zebrafish

To determine appropriate exposure concentrations for subsequent bioconcentration testing while minimizing abnormal behavior and mortality, it was necessary to establish the LC_50_ value for zebrafish. In a previous study, LC_50_ values for α-cypermethrin in zebrafish were reported as 1.29 µg/L at 24 h, 0.64 µg/L at 48 h, 0.29 µg/L at 72 h, and 0.17 µg/L at 96 h. In contrast, another prior study reported no mortality in zebrafish exposed to 3 µg/L cypermethrin for eight days, indicating considerable inter-study variability.

To obtain more precise toxicity thresholds under the present experimental conditions, an acute toxicity test was conducted. Consistent with previous findings, zebrafish exposed to α-cypermethrin exhibited reduced and irregular swimming behavior, and LC_50_ values decreased with longer exposure durations. As shown in Table 1, the LC_50_ values were determined to be 1.54 µg/L (95% confidence interval: 1.16–2.05 µg/L) at 24 and 48 h, and 1.28 µg/L (95% confidence interval: 1.07–1.54 µg/L) at 72 and 96 h. Based on this result, exposure concentrations of 0.15 µg/L and 1.5 µg/L, and exposure durations of 24 h and 48 h, were selected for exposure scenarios. No mortality was observed in either exposure group during the 48 h treatment period, indicating that both concentrations were within the sublethal range.

### 3.2. Exposure Concentration Verification

To monitor the concentration stability of α-cypermethrin during the exposure period, 2 mL water samples were collected from the exposure tanks corresponding to the low (0.15 µg/L) and high (1.5 µg/L) concentrations at 0, 24, and 48 h. A six-point calibration curve ranging from 0.075 to 2.0 µg/L was constructed. The calibration curve exhibited excellent linearity, with an R^2^ value of 0.999 (Figure S1).

As summarized in Table 2, the measured concentrations of α-cypermethrin remained consistent throughout the exposure period. Concentrations ranged from 0.138 to 0.147 µg/L in the low-concentration group and from 1.42 to 1.47 µg/L in the high-concentration group. These results indicate minimal variation in exposure levels over time.

### 3.3. Metabolomic Alternation Induced by α-Cypermethrin

**Figure 1:**
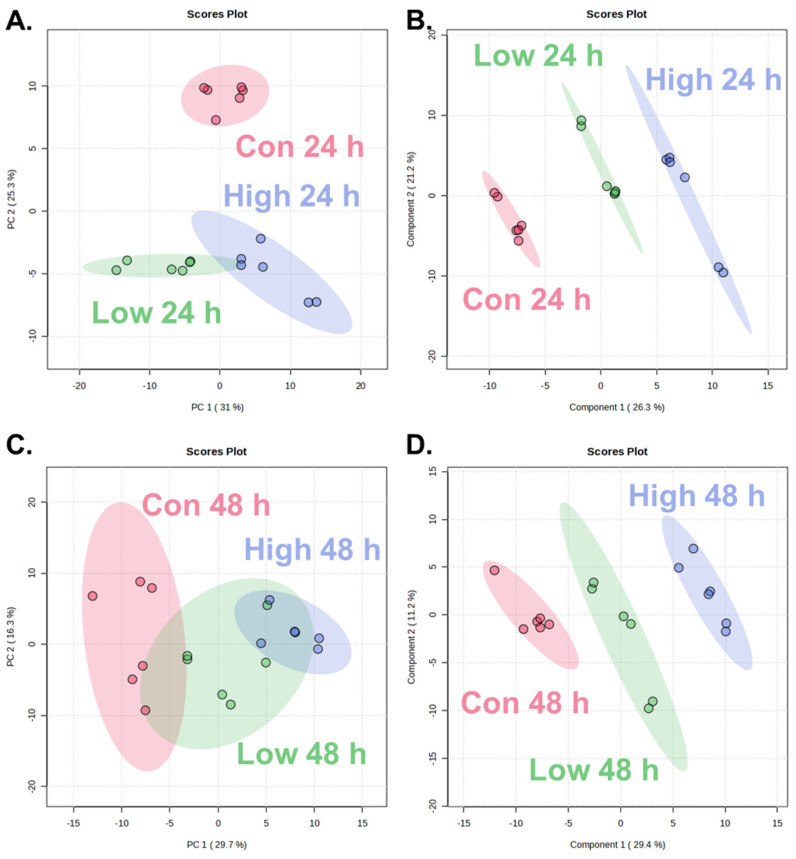
Score plots of multivariate analysis showing metabolic changes in zebrafish after α-cypermethrin exposure: at 24 h post-exposure, PCA plot (**A**) and PLS-DA plot (**B**), and at 48 h post-exposure, PCA plot (**C**) and PLS-DA plot (**D**).

To evaluate the time-dependent and dose-dependent metabolic alterations following α-cypermethrin exposure, PCA and PLS-DA were conducted using the metabolite profiles of zebrafish collected at 24 and 48 h post-treatment (Figure 1).

At 24 h post-exposure, the PCA score plot (Figure 1A) revealed clear separation between the control group (Con 24 h) and both treatment groups along PC2 (25.3%). Although the low- and high-dose groups exhibited partial overlap, a dose-dependent shift along PC1 (31.0%) was observed. This separation was further confirmed by the PLS-DA model (Figure 1B). All three groups were discriminated along the two components (Component 1: 26.3%; Component 2: 21.2%).

At 48 h post-exposure, PCA revealed partial separation among the control, low-dose, and high-dose groups (Figure 1C), with a clear distinction between the control and high-dose groups along PC1 (29.7%). The low-dose group overlapped partially with both control and high-dose groups, indicating a moderate metabolic shift. PLS-DA revealed clear separation among the control, low-dose, and high-dose groups (Figure 1D), with a pronounced dose-dependent distribution along Component 1 (29.4%) and Component 2 (11.2%). Compared to the PCA model which showed partial overlap between the low- and high-dose groups, PLS-DA provided improved class discrimination.

A consistent dose-dependent separation was observed at both time points in both PCA and PLS-DA models. The high-dose groups were positioned farther from the control than low-dose groups. This spatial divergence reflected the extent of metabolic changes, indicating that higher concentrations of α-cypermethrin lead to more pronounced biochemical alterations. These findings demonstrate a clear concentration-dependent response at the metabolome level.

The reliability and predictive power of the PLS-DA models were assessed using R^2^ and Q^2^ values. At 24 h post-exposure, the model showed excellent performance with R^2^ = 0.9839 and Q^2^ = 0.9415, while the 48 h model also exhibited robust validity with R^2^ = 0.9815 and Q^2^ = 0.9147. Both Q^2^ values exceeded the commonly accepted threshold of 0.4 for robust models, confirming that the observed group separations were statistically reliable and highly predictive [34,35,36]. These results support the suitability of each PLS-DA model for identifying dose-dependent metabolic alterations induced by α-cypermethrin.

Given that the high-dose groups exhibited greater separation from the control than the low-dose groups in the PLS-DA models, subsequent analyses focused on identifying significantly altered metabolites between the control and high-dose groups at each time point. Based on univariate *t*-tests (*p* < 0.05), 79 and 62 significant metabolites were identified at 24 and 48 h post-exposure, respectively (Tables S3 and S4).

**Figure 2:**
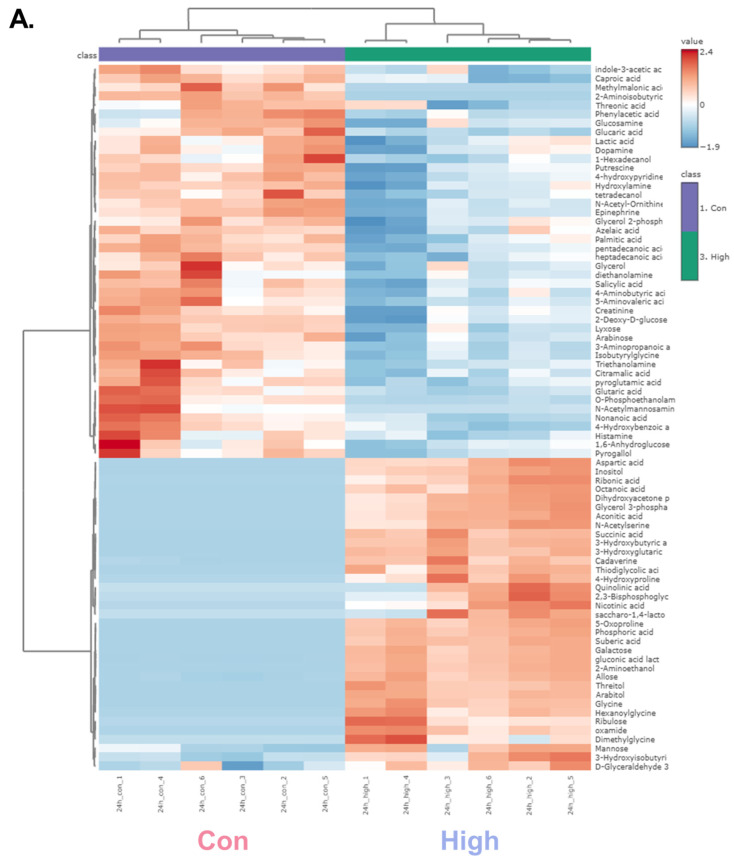

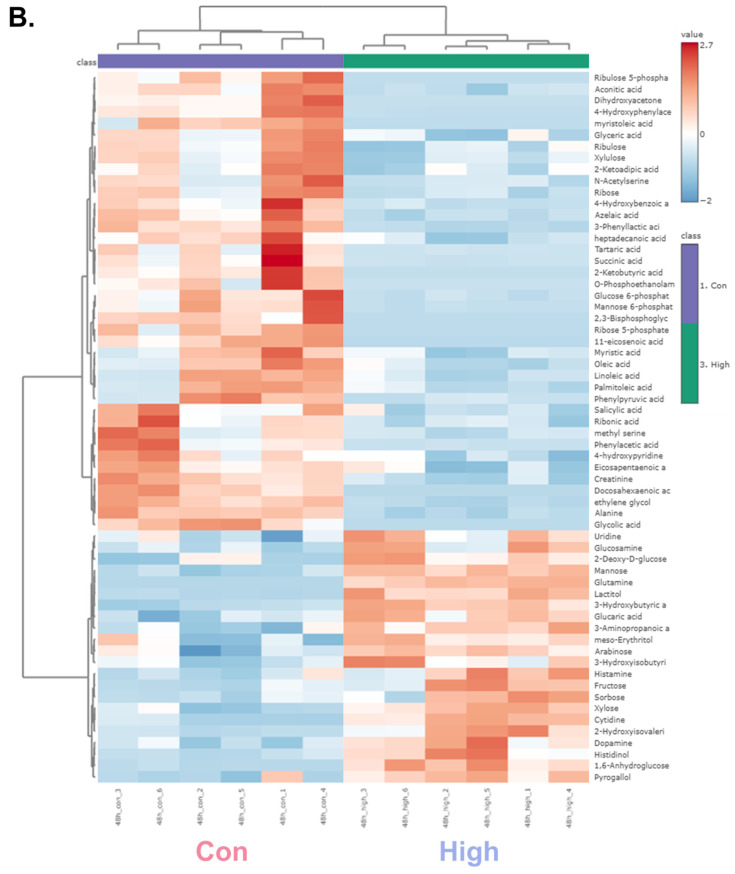
Hierarchical clustering heatmaps of significantly altered metabolites in zebrafish following high-dose α-cypermethrin exposure: (**A**) 24 h and (**B**) 48 h exposure groups.

A hierarchical clustering heatmap was generated to visualize the metabolic profiles of zebrafish at 24 and 48 h post-exposure (Figure 2). The color indicated the levels of metabolite changes from highest (red) to lowest (blue). At both 24 and 48 h post-exposure groups, the heatmap revealed clear clustering between the control and high-dose groups. Notably, glycolysis-related metabolites and long-chain fatty acids were markedly changed. In the 24 h group, metabolites such as glycerol, D-glyceraldehyde 3-phosphate, and dihydroxyacetone showed marked alterations, whereas in the 48 h group, significant changes were observed in glucose-6-phosphate and mannose-6-phosphate. Long-chain fatty acids were decreased in both the 24 and 48 h groups, indicating a possible link to changes in energy metabolism [37]. Collectively, the altered patterns of carbohydrate intermediates and lipid metabolites point to α-cypermethrin-induced perturbations in central energy metabolism.

### 3.4. Metabolic Pathway Analysis

**Figure 3:**
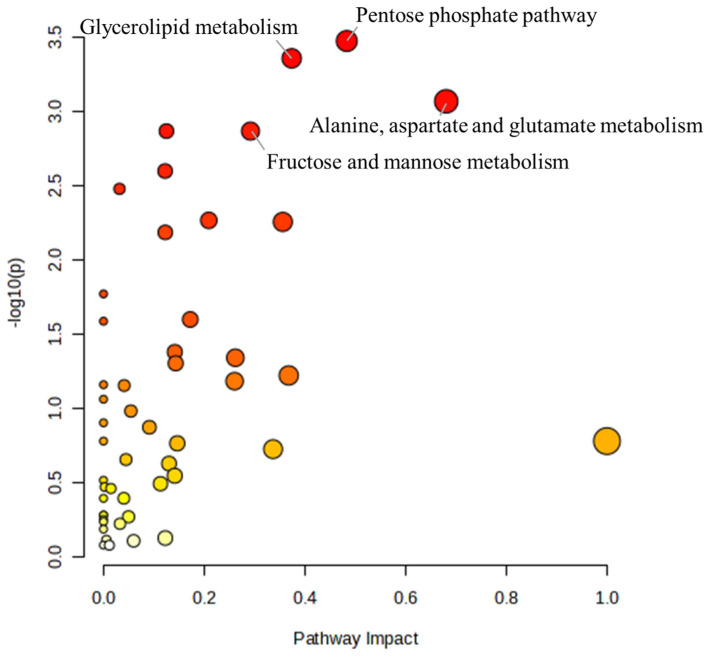
Bubble plot summarizing the metabolic pathway analysis conducted using MetaboAnalyst 6.0. Bubble size indicates pathway impact, and color intensity reflects statistical significance.

To further understand the effects of α-cypermethrin on zebrafish, the pathway analysis was conducted. A total of 114 metabolites were identified as significantly altered (*p* < 0.05) at either the 24 h or 48 h groups and were selected for pathway analysis. The results of the pathway analysis are presented as a bubble plot in Figure 3. The size of each bubble reflects the pathway impact score, while the color gradient represents the level of statistical significance, ranging from the most significant (red) to the least (white) [38]. Based on this result, four metabolic pathways were identified as major disturbed pathways, namely, the pentose phosphate pathway, glycerolipid metabolism, alanine/aspartate/glutamate metabolism, and fructose and mannose metabolism.

**Figure 4:**
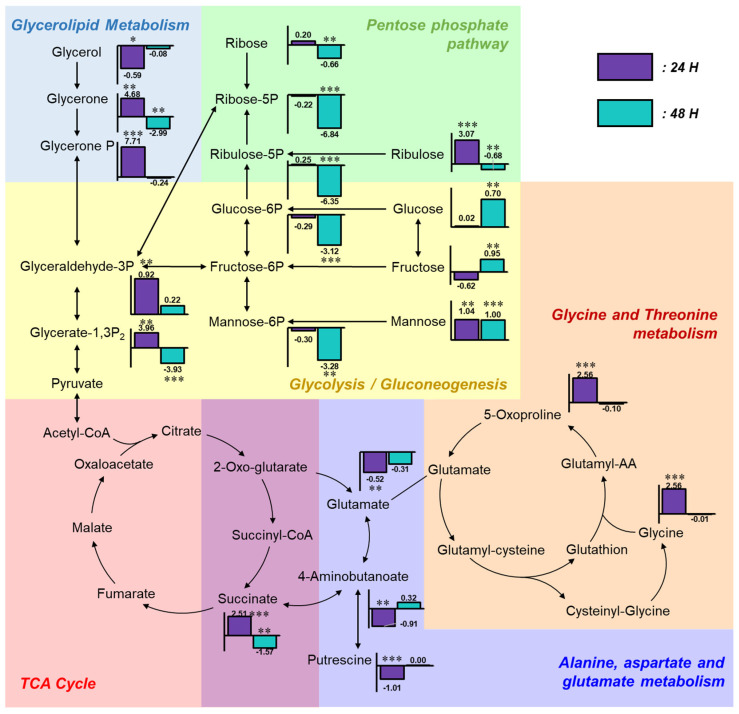
Integrated metabolic pathway map illustrating fold changes in key metabolites following α-cypermethrin exposure. Bar plots represent log_2_ fold changes relative to the control group at 24 h (purple) and 48 h (blue), with asterisks indicating statistical significance (* *p* < 0.05; ** *p* < 0.01; *** *p* < 0.001).

To investigate the relationships among altered metabolites, a metabolic pathway map was constructed and annotated with fold change values of each compound relative to the control group (Figure 4). In the glycolysis region of the metabolic pathway map, the levels of glucose and fructose remained unchanged at 24 h and became markedly elevated at 48 h post-exposure, indicating accumulation in response to α-cypermethrin. Mannose levels were consistently increased at both time points. However, phosphorylated sugars such as glucose-6-phosphate and mannose-6-phosphate were significantly decreased at 48 h. This difference between free sugars and their phosphorylated counterparts suggested a metabolic bottleneck at the phosphorylation step. This result suggested that carbon flux into glycolysis is constrained at the phosphorylation steps. Despite this limitation, most TCA cycle intermediates showed no statistically significant differences compared to the control group, except for succinic acid, indicating that carbon entry into the TCA cycle originates from non-sugar sources.

Four metabolites involved in glycolysis and glycerolipid metabolism (glycerone, glycerone phosphate, glyceraldehyde-3-phosphate, and glycerate-1,3-bisphosphate) were markedly increased at 24 h post-α-cypermethrin exposure. However, these metabolites exhibited diminished fold changes or were downregulated at 48 h compared to controls. This temporal pattern, along with a consistent reduction in long-chain fatty acids, suggested the mobilization of fatty acids as an alternative energy source under α-cypermethrin-induced stress, possibly compensating for impaired glycolytic input.

This interpretation is supported by a previous study in *Ophiocephalus punctatus*, where cypermethrin exposure led to a significant decrease in total lipid content in liver and intestinal tissues, indicating that lipid reserves were likely catabolized to meet elevated energy demands during toxicant-induced metabolic stress.

Among amino acid-related metabolites, glycine, glutamate, and 5-oxoproline showed significant alterations at 24 h post-pesticide exposure compared to the control group. These compounds are closely associated with the glutathione metabolism pathway [39]. Under typical xenobiotic stress conditions, the synthesis of glutathione (GSH) is upregulated to counteract increased oxidative burden [40,41]. According to a previous study, cypermethrin can induce oxidative stress in adult zebrafish [17]. However, the observed accumulation of 5-oxoproline alongside the depletion of glutamate implies the presence of a metabolic bottleneck at the step catalyzed by 5-oxoprolinase, which converts 5-oxoproline to glutamate. This bottleneck likely limited the availability of glutamate, not only restricting glutathione regeneration but also affecting other glutamate-dependent pathways.

One such affected pathway is the GABA shunt, where 4-aminobutanoate (GABA) is synthesized from glutamate [42]. The observed reduction in GABA is most likely attributable to the decreased availability of glutamate, which serves as its direct precursor via glutamate decarboxylase [43]. As GABA is involved in multiple physiological processes beyond neurotransmission, including the modulation of intracellular redox status, the regulation of osmotic balance, and functioning as a signaling molecule in stress adaptation, its depletion under α-cypermethrin-induced stress may compromise cellular homeostasis [42,44].

Despite continuous chemical exposure, the levels of 5-oxoproline, GABA, and glutamate were not significantly different from those in the control group at 48 h post-exposure. This metabolic normalization suggested that zebrafish underwent adaptive reprogramming to tolerate sustained α-cypermethrin-induced stress. Especially, the recovery of glutamate-related compounds reflects a stabilization of amino acid metabolism, contributing to redox regulation and supporting overall metabolic homeostasis under toxic conditions.

## 4. Conclusions

This study employed a targeted metabolomics approach to investigate the metabolic effects of sublethal α-cypermethrin exposure in adult zebrafish over two time points (24 h and 48 h). Time- and dose-dependent alterations were observed in several key pathways, including glycolysis, glycerolipid metabolism, and glutathione turnover. At 24 h post-exposure, marked increases were observed in glycolytic intermediates such as glyceraldehyde-3-phosphate and glycerone phosphate, while 5-oxoproline accumulation and glutamate depletion indicated early disturbances in glutathione metabolism. At 48 h, a consistent decrease in long-chain fatty acids and phosphorylated sugars such as glucose-6-phosphate and mannose-6-phosphate suggested sustained oxidative stress and impaired energy metabolism. The reduction in GABA at 24 h further implied the downstream disruption of the TCA cycle via glutamate depletion. These findings demonstrate that α-cypermethrin triggers early metabolic bottlenecks that propagate across interconnected biochemical pathways, ultimately affecting redox balance and central carbon metabolism. Alterations in glutathione-related pathways observed in this study suggest a disruption of redox homeostasis, highlighting the need for future investigations focused on oxidative stress responses and antioxidant defense mechanisms in pesticide-exposed aquatic organisms. Although this study focused on metabolic alterations, the integration of phenotypic assessments—such as behavioral or physiological endpoints—would further strengthen the interpretation of sublethal effects. This study provides a systems-level perspective on pesticide-induced metabolic stress and supports the use of metabolomic indicators for environmental monitoring and risk evaluation. Future multi-omics approaches will be valuable to further elucidate the regulatory mechanisms underlying these metabolic shifts.

## Figures and Tables

**Table 1:** Toxicity of α-cypermethrin against zebrafish.

Exposure Time	24 h	48 h	72 h	96 h
LC_50_ (µg/L)	1.54	1.54	1.28	1.28
95% Confidence limits (µg/L)	1.16–2.05	1.16–2.05	1.07–1.54	1.07–1.54

**Table 2:** Concentration changes over exposure time at low concentration (0.15 µg/L) and high concentration (1.5 µg/L).

Exposure Time	0.15 µg/L		1.5 µg/L	
	Conc.(µg/L)	Ratio (%) *	Conc. (µg/L)	Ratio (%) *
0 h	0.147	98	1.47	98
24 h	0.140	93	1.44	96
48 h	0.138	92	1.42	95

* Ratio (%): the proportion of the measured concentration relative to the calibration concentration.

## Data Availability

The raw data supporting the conclusions of this article will be made available by the authors upon request.

## Supplementary Materials

The following supporting information can be downloaded at: https://www.mdpi.com/article/10.3390/toxics13070529/s1, Figure S1: Calibration curve of α-cypermethrin in water samples at six concentration points; Figure S2: Performance metrics (accuracy, R^2^, and Q^2^) from PLS-DA cross-validation at 24 h and 48 h; Table S1: Analytical conditions of LC-MS/MS for α-cypermethrin; Table S2: Multiple reaction monitoring transitions and retention times for quantitative analysis of α-cypermethrin in zebrafish; Table S3: Significantly altered metabolites between the control and high-dose groups at 24 h post-exposure, identified by a univariate *t*-test (*p* < 0.05); Table S4: Significantly altered metabolites between the control and high-dose groups at 48 h post-exposure, identified by a univariate *t*-test (*p* < 0.05).

## Abbreviations

The following abbreviations are used in this manuscript:

MSTFA	*N*-methyl-*N*-(trimethylsilyl)trifluoroacetamide reagent containing 1% trimethylsilyl chloride
UHPLC–MS/MS	Ultra-high performance liquid chromatography tandem mass spectrometry
MRM	Multiple reaction monitoring
PCA	Principal component analysis
PLS-DA	Partial least squares-discriminant analysis
GABA	4-Aminobutanoate
