# Supplementary material for: Metabolic Disruptions in Zebrafish Induced by α-Cypermethrin: A Targeted Metabolomics Study
Source: Toxics. 2025 Jun 24;13(7):529. doi: 10.3390/toxics13070529 (PMC12300848; doi:10.3390/toxics13070529)
Supplement: Supplementary file 1 [file toxics-13-00529-s001.zip › toxics-3692692-supplementary.pdf]

Article

Metabolic Disruptions in Zebrafish Induced by α-Cypermethrin: A Targeted Metabolomics Study

Hang-Ji Ok[1,2†], Ji-Woo Yu[3†], Jung-Hoon Lee[4,5], Eun-Song Choi[4,5], Jong-Hwan Kim[6, 7], Yoonjeong Jeon[6], Won Noh[6], Sung-Gil Choi[6], Jeong-Han Kim[1], Min-Ho Song[4*], and Ji-Ho Lee[4,5*]

[1]*Department of Agricultural Biotechnology of Agriculture and Life Sciences, Seoul National University, Seoul, Republic of Korea*

[2]*Department of Technical Research Center, Shimadzu Scientific Korea, Seoul, Republic of Korea*

[3]*Department of Crop Science, Konkuk University, 120 Neungdong-ro, Gwangjin-gu, Seoul 05029, Republic of Korea*

[4]*School of Natural Resources and Environment Science College of Agriculture and Life Sciences, Kangwon National University, Gangwon State 24341, Republic of Korea*

[5]*Department of Food Biotechnology and Environmental Science, Kangwon National University, Chuncheon, Gangwon State, 24341, Republic of Korea*

[6]*Environmental Safety Research Center, Korea Institute of Toxicology (KIT), Jinju 52834, Republic of Korea.*

[7]*Human and Environmental Toxicology Program, Korea University of Science and Technology (UST), Deajeon, Republic of Korea*

[†]These authors contributed equally.

*Corresponding author:

*Min-Ho Song

E-mail: minobel@naver.com

Phone No.: +82-10-4731-6187

Fax: +82-33-259-5563

*Ji-Ho Lee

E-mail: micai@kangwon.ac.kr

Phone No.: +82-10-2012-5548

Fax: +82-33-259-5563

Table S1. Analytical conditions of LC-MS/MS for α-cypermethrin

**UHPLC**	**Nexera XS system**
Mobile phase A	: 5 mM Ammonium acetate, 0.1 % formic acid in water
Mobile phase B	: 5 mM Ammonium acetate, 0.1 % formic acid in MeOH
Flow rate	: 0.3 mL/min
Gradient	: B 5 % (0.0 - 0.5 min) – B 60 % (1.5 min) – B 100 % (6.0 min) - B 100 % (8.0 min) – B 5 % (8.1 min) – B 5 % (10 min)
Analytical column	: Shim-pack GISS C18 (2.1 x 50 mm, 1.9 μm)
Column oven temp.	: 40℃
Injection volume	: 50 μL
**MS/MS**	**LCMS-8060NX**
Ionization method	: ESI Positive
Nebulizing gas flow	: 3 L/min
Heating gas flow	: 15 L/min
Drying gas temp.	: 3 L/min
Interface temp.	: 200℃
DL temp.	: 255℃
Heat block temp.	: 400℃

Table S2. Multiple reaction monitoring transitions and retention times for quantitative analysis of α-cypermethrin in zebrafish.

Pesticide	Retention time (min)	Precursor ion (m/z)	Quantitation ion (m/z) (CE*, eV) / Qualification ion (m/z) (CE*, eV)
α-cypermethrin	5.676	433	191 (-17)
			416 (-9)

Table S3. Significantly altered metabolites between the control and high-dose groups at 24 hours post-exposure, identified by univariate t-test ($P < 0.05$).

Compound	t.stat	p.value	-LOG10(p)	FDR
3-Hydroxyglutaric acid	-25.54	2.E-10	9.71	1.E-08
Suberic acid	-24.89	3.E-10	9.60	1.E-08
2-Aminoisobutyric acid	24.53	3.E-10	9.54	1.E-08
2-Aminoethanol	-24.43	3.E-10	9.52	1.E-08
Phosphoric acid	-23.99	4.E-10	9.44	1.E-08
gluconic acid lactone	-23.29	5.E-10	9.32	1.E-08
3-Hydroxybutyric acid	-21.75	9.E-10	9.03	2.E-08
Arabitol	-21.35	1.E-09	8.95	3.E-08
Galactose	-20.55	2.E-09	8.78	3.E-08
Allose	-19.04	3.E-09	8.46	6.E-08
5-Oxoproline	-18.40	5.E-09	8.32	8.E-08
Threitol	-16.39	1.E-08	7.83	2.E-07
Succinic acid	-15.79	2.E-08	7.67	3.E-07
Glycine	-15.76	2.E-08	7.66	3.E-07
Inositol	-13.14	1.E-07	6.91	2.E-06
Octanoic acid	-11.87	3.E-07	6.49	4.E-06
Aconitic acid	-11.31	5.E-07	6.29	5.E-06
Thiodiglycolic acid	-11.24	5.E-07	6.27	5.E-06
Cadaverine	-11.21	6.E-07	6.26	5.E-06
Glycerol 3-phosphate	-10.47	1.E-06	5.98	1.E-05
Dihydroxyacetone phosphate	-10.18	1.E-06	5.87	1.E-05
Aspartic acid	-9.93	2.E-06	5.77	1.E-05
N-Acetylserine	-9.53	2.E-06	5.61	2.E-05
Hexanoylglycine	-9.49	3.E-06	5.59	2.E-05
oxamide	-8.87	5.E-06	5.33	3.E-05
Ribonic acid	-8.20	9.E-06	5.03	7.E-05
4-Hydroxyproline	-7.29	3.E-05	4.58	2.E-04
Methylmalonic acid	6.79	5.E-05	4.32	3.E-04
Isobutyrylglycine	6.74	5.E-05	4.29	3.E-04
Epinephrine	6.53	7.E-05	4.18	4.E-04
Glucaric acid	6.38	8.E-05	4.09	5.E-04
pentadecanoic acid	6.25	9.E-05	4.02	5.E-04
3-Aminopropanoic acid	6.20	1.E-04	4.00	6.E-04
N-Acetyl-Ornithine	5.95	1.E-04	3.85	8.E-04
Caproic acid	5.91	1.E-04	3.82	8.E-04
Arabinose	5.77	2.E-04	3.74	9.E-04
Lyxose	5.75	2.E-04	3.73	9.E-04
4-hydroxypyridine	5.61	2.E-04	3.65	1.E-03
Ribulose	-5.47	3.E-04	3.56	1.E-03
Nicotinic acid	-5.35	3.E-04	3.49	1.E-03
O-Phosphoethanolamine	5.15	4.E-04	3.36	2.E-03

Compound	t.stat	p.value	-LOG10(p)	FDR
4-Hydroxybenzoic acid	5.05	5.E-04	3.30	2.E-03
3-Hydroxyisobutyric acid	-5.05	5.E-04	3.30	2.E-03
heptadecanoic acid	4.97	6.E-04	3.25	2.E-03
2-Deoxy-D-glucose	4.84	7.E-04	3.17	3.E-03
5-Aminovaleric acid	4.74	8.E-04	3.10	3.E-03
pyroglutamic acid	4.70	8.E-04	3.07	3.E-03
Nonanoic acid	4.64	9.E-04	3.03	4.E-03
Putrescine	4.54	1.E-03	2.97	4.E-03
tetradecanol	4.50	1.E-03	2.94	4.E-03
Palmitic acid	4.49	1.E-03	2.94	4.E-03
Hydroxylamine	4.49	1.E-03	2.93	4.E-03
Dihydroxyacetone	4.38	1.E-03	2.86	5.E-03
Glutaric acid	4.38	1.E-03	2.86	5.E-03
Pyrogallol	4.27	2.E-03	2.79	5.E-03
4-Aminobutyric acid	4.22	2.E-03	2.75	6.E-03
Citramalic acid	4.12	2.E-03	2.68	7.E-03
Salicylic acid	4.09	2.E-03	2.66	7.E-03
Mannose	-3.94	3.E-03	2.56	9.E-03
1,3-Bisphosphoglyceric acid	-3.92	3.E-03	2.54	9.E-03
1-Hexadecanol	3.71	4.E-03	2.39	1.E-02
Triethanolamine	3.67	4.E-03	2.36	1.E-02
Dimethylglycine	-3.58	5.E-03	2.30	1.E-02
D-Glyceraldehyde 3-phosphate	-3.56	5.E-03	2.29	1.E-02
Creatinine	3.53	5.E-03	2.26	2.E-02
Azelaic acid	3.49	6.E-03	2.23	2.E-02
saccharo-1,4-lactone	-3.32	8.E-03	2.11	2.E-02
Glucosamine	3.26	9.E-03	2.06	2.E-02
Histamine	3.19	1.E-02	2.01	3.E-02
Phenylacetic acid	3.13	1.E-02	1.97	3.E-02
Glycerol 3-phosphate	3.13	1.E-02	1.97	3.E-02
Quinolinic acid	-3.09	1.E-02	1.94	3.E-02
diethanolamine	3.06	1.E-02	1.92	3.E-02
Dopamine	3.06	1.E-02	1.92	3.E-02
N-Acetylmannosamine	3.01	1.E-02	1.89	3.E-02
Glycerol	2.96	1.E-02	1.84	3.E-02
Lactic acid	2.89	2.E-02	1.79	4.E-02
Threonic acid	2.88	2.E-02	1.79	4.E-02
1,6-Anhydroglucose	2.78	2.E-02	1.71	5.E-02

Table S4. Significantly altered metabolites between the control and high-dose groups at 48 hours post-exposure, identified by univariate t-test (P < 0.05).

Compound	t.stat	p.value	-LOG10(p)	FDR
Glutamine	-19.10	3.E-09	8.47	6.E-07
Alanine	12.78	2.E-07	6.79	1.E-05
Mannose	-12.61	2.E-07	6.74	1.E-05
ethylene glycol	11.86	3.E-07	6.49	1.E-05
3-Hydroxybutyric acid	-10.48	1.E-06	5.99	4.E-05
Lactitol	-10.13	1.E-06	5.85	4.E-05
Docosahexaenoic acid	8.46	7.E-06	5.14	2.E-04
3-Phenyllactic acid	8.05	1.E-05	4.95	3.E-04
Cytidine	-7.63	2.E-05	4.75	4.E-04
11-eicosenoic acid	7.52	2.E-05	4.69	4.E-04
1,6-Anhydroglucose	-7.17	3.E-05	4.52	5.E-04
Creatinine	6.91	4.E-05	4.38	6.E-04
Glycolic acid	6.58	6.E-05	4.20	9.E-04
Ribose 5-phosphate	6.03	1.E-04	3.89	2.E-03
Xylose	-5.50	3.E-04	3.58	3.E-03
1,3-Bisphosphoglyceric acid	5.49	3.E-04	3.57	3.E-03
myristoleic acid	5.37	3.E-04	3.50	3.E-03
Azelaic acid	5.12	5.E-04	3.35	5.E-03
Glucaric acid	-5.09	5.E-04	3.33	5.E-03
Aconitic acid	4.95	6.E-04	3.24	5.E-03
3-Aminopropanoic acid	-4.69	9.E-04	3.07	7.E-03
Ribulose 5-phosphate	4.62	9.E-04	3.02	8.E-03
4-Hydroxyphenylacetic acid	4.53	1.E-03	2.96	9.E-03
2-Hydroxyisovaleric acid	-4.49	1.E-03	2.94	9.E-03
Histidinol	-4.41	1.E-03	2.88	1.E-02
2-Ketobutyric acid	4.36	1.E-03	2.85	1.E-02
Mannose 6-phosphate	4.17	2.E-03	2.72	1.E-02
Dopamine	-4.16	2.E-03	2.71	1.E-02
Glucose 6-phosphate	4.02	2.E-03	2.61	2.E-02
Pyrogallol	-3.99	3.E-03	2.59	2.E-02
Dihydroxyacetone	3.94	3.E-03	2.56	2.E-02
Ribose	3.82	3.E-03	2.47	2.E-02
Xylulose	3.71	4.E-03	2.39	2.E-02
heptadecanoic acid	3.68	4.E-03	2.37	2.E-02
2-Deoxy-D-glucose	-3.63	5.E-03	2.34	2.E-02
4-Hydroxybenzoic acid	3.59	5.E-03	2.31	2.E-02
Arabinose	-3.58	5.E-03	2.30	2.E-02
Ribonic acid	3.58	5.E-03	2.30	2.E-02
Glucosamine	-3.56	5.E-03	2.28	2.E-02
Ribulose	3.52	6.E-03	2.25	3.E-02
Eicosapentaenoic acid	3.46	6.E-03	2.21	3.E-02
Fructose	-3.45	6.E-03	2.21	3.E-02
Phenylacetic acid	3.45	6.E-03	2.21	3.E-02
O-Phosphoethanolamine	3.31	8.E-03	2.10	3.E-02
Glucose	3.29	8.E-03	2.09	3.E-02
Salicylic acid	3.27	8.E-03	2.07	3.E-02

Compound	t.stat	p.value	-LOG10(p)	FDR
Palmitoleic acid	3.26	9.E-03	2.07	3.E-02
Oleic acid	3.24	9.E-03	2.05	3.E-02
Ribitol	3.23	9.E-03	2.05	3.E-02
Phenylpyruvic acid	3.23	9.E-03	2.04	3.E-02
Succinic acid	3.16	1.E-02	2.00	4.E-02
Sorbose	-3.16	1.E-02	1.99	4.E-02
methyl serine	3.13	1.E-02	1.97	4.E-02
Linoleic acid	3.11	1.E-02	1.96	4.E-02
3-Hydroxyisobutyric acid	-3.08	1.E-02	1.93	4.E-02
Glyceric acid	3.01	1.E-02	1.88	4.E-02
Uridine	-2.95	1.E-02	1.84	5.E-02
N-Acetylserine	2.95	1.E-02	1.84	5.E-02
Myristic acid	2.95	1.E-02	1.83	5.E-02
Tartaric acid	2.92	2.E-02	1.82	5.E-02
Histamine	-2.90	2.E-02	1.80	5.E-02
2-Ketoadipic acid	2.89	2.E-02	1.79	5.E-02
meso-Erythritol	-2.87	2.E-02	1.78	5.E-02

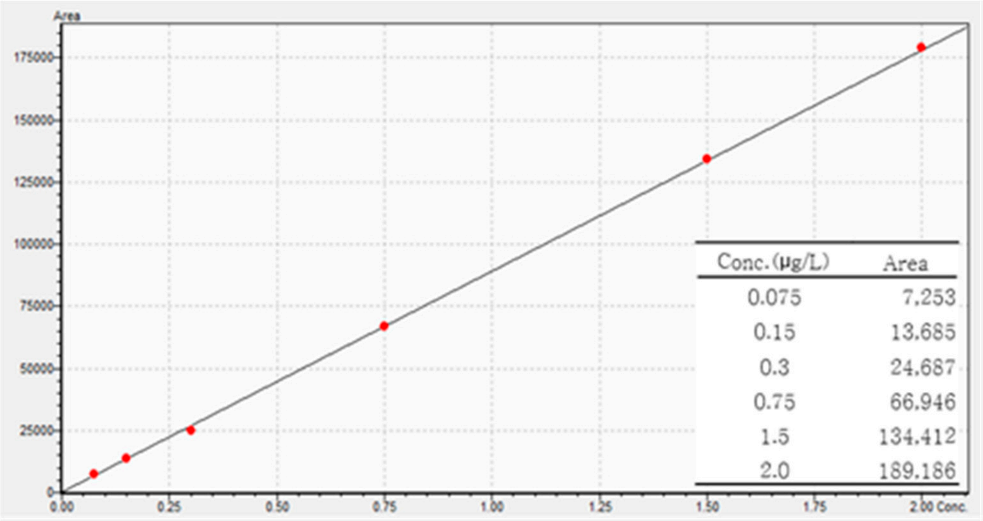

Figure S1. Calibration curve of α-cypermethrin in water samples at six concentration points.

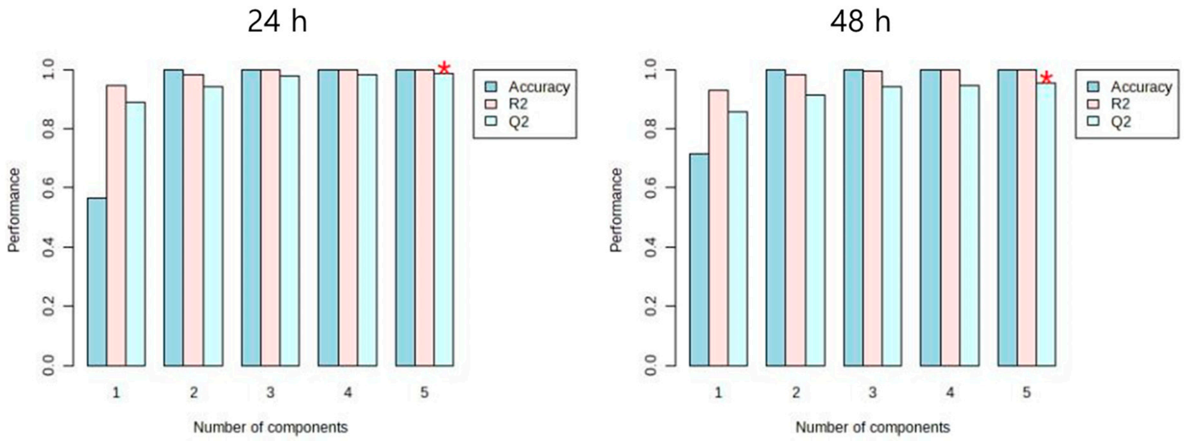

Figure S2. Performance metrics (accuracy, R^2, Q^2) from PLS-DA cross-validation at 24 h and 48 h.
